# Dysfunctional Mitochondria Characterize Amyotrophic Lateral Sclerosis Patients’ Cells Carrying the p.G376D *TARDBP* Pathogenetic Substitution

**DOI:** 10.3390/antiox14040401

**Published:** 2025-03-28

**Authors:** Giuseppe Petito, Victoria Stefania Del Fiore, Arianna Cuomo, Federica Cioffi, Gilda Cobellis, Antonia Lanni, Flora Guerra, Cecilia Bucci, Rosalba Senese, Roberta Romano

**Affiliations:** 1 Department of Environmental, Biological and Pharmaceutical Sciences and Technologies, University of Campania “Luigi Vanvitelli”, 81100 Caserta, Italy; giuseppe.petito@unicampania.it (G.P.); arianna.cuomo@unicampania.it (A.C.); antonia.lanni@unicampania.it (A.L.); rosalba.senese@unicampania.it (R.S.); 2 Department of Experimental Medicine (DiMeS), University of Salento, Via Provinciale Lecce-Monteroni n.165, 73100 Lecce, Italy; victoriastefania.delfiore@unisalento.it (V.S.D.F.); roberta.romano@unisalento.it (R.R.); 3 Department of Sciences and Technologies, University of Sannio, 82100 Benevento, Italy; federica.cioffi@unisannio.it; 4 Department of Experimental Medicine, University of Campania “Luigi Vanvitelli”, 80138 Naples, Italy; gilda.cobellis@unicampania.it; 5 Department of Biological and Environmental Sciences and Technologies (DiSTeBA), University of Salento, Via Provinciale Lecce-Monteroni n.165, 73100 Lecce, Italy; flora.guerra@unisalento.it

**Keywords:** amyotrophic lateral sclerosis, TDP-43, mitochondria, oxidative stress, TARDBP

## Abstract

Amyotrophic lateral sclerosis (ALS) is a neurodegenerative disease caused by the degeneration of upper and lower motor neurons in the brain, brainstem and spinal cord. About 10% of familial ALS cases are linked to pathogenetic substitution in *TARDBP*, the gene encoding the TDP-43 protein. A novel rare causative variant in *TARDBP* (p.G376D) was recently reported in ALS patients. It leads to TDP-43 cytoplasmic mislocalization, increased oxidative stress and reduced cell viability. However, functional studies on the effects of this molecular defect have not yet been carried out. Mitochondria are highly dynamic organelles, and their deregulation has emerged as a key factor in many diseases, among which is ALS. Therefore, this study aimed at determining the impact of this causative variant on mitochondria. In cellular models expressing TDP-43^G376D^ and in fibroblasts derived from patients carrying this molecular defect, we observed alterations of mitochondrial functionality. We demonstrated increased localization of the mutated protein to mitochondria and a reduced abundance of subunits of complex I and complex II of the mitochondrial respiratory chain, associated with a decrease in mitochondrial membrane potential, in cellular respiration and in cytochrome C oxidase (COX) activity. Moreover, ALS cells showed increased mitochondrial fragmentation and reduced abundance of antioxidant enzymes causing increased oxidative stress. These results expand our knowledge about the molecular mechanisms underlying ALS pathogenesis associated with TDP-43 p.G376D and could help to identify new therapeutic strategies to counteract this disease.

## 1. Introduction

Amyotrophic lateral sclerosis (ALS) is a neurodegenerative disease caused by the degeneration of motor neurons in the brain, brainstem, and spinal cord [1,2]. Adult-onset focal muscle weakness and wasting are early symptoms of ALS with a tendency to spread with disease progression. Unfortunately, this progression leads to the loss of the ability to breathe without medical assistance, and death occurs about three years after symptom manifestation [3,4]. ALS can be sporadic or familial: the sporadic form includes about 90% of ALS cases, and it is characterized by the absence of prior family history, while the familial form (the remaining 10% of ALS cases) is caused by pathogenetic variants with an autosomal dominant inheritance pattern [3,5,6].

Alterations in more than 40 genes have been identified as causative of ALS [7,8]. Among familial ALS cases, about 10% are caused by pathogenetic substitution in the *TARDBP* gene, coding for TDP-43 (Transactive response DNA binding protein of 43 kDa), and an RNA/DNA binding protein involved in RNA metabolism [7]. Being able to shuttle between the nucleus and the cytoplasm, TDP-43 has a nuclear localization signal in its N-terminal region and a nuclear export signal [9]. More than 50 causative variants in the *TARDBP* gene have been associated with ALS pathogenesis, highlighting the importance of TDP-43 alterations in ALS onset [10].

TDP-43 aggregates represent a pathological hallmark of ALS. Indeed, in 2006, this protein was detected in the insoluble and ubiquitinated aggregates in the brains of patients [11,12]. Wild-type TDP-43 was also found in the inclusions of most ALS patients, suggesting that alterations in the levels or localization of wild-type TDP-43 are linked to both sporadic and familial ALS [11]. ALS-causing pathogenetic substitutions are associated with increased aggregation, enhanced cytoplasmic mislocalization, and altered protein stability [13]. Most of these causative variants are localized in the C-terminal domain of TDP-43, enhancing its intrinsic aggregation propensity [9,14]. The most common and the most studied TDP-43 causative variants are A382T and M337V [15], while the less characterized G376D pathogenetic substitution is responsible for a rare familial ALS reported in South Italy, Switzerland, and Japan [16,17,18]. TDP-43^G376D^ showed reduced nuclear localization and enhanced cytoplasmic mislocalization, leading to the appearance of cytoplasmic aggregates [17,19].

Several pathways have been found to be compromised in ALS: increased oxidative stress, defective transport in the axons, excitotoxicity, protein aggregation, endoplasmic reticulum stress, and mitochondrial dysfunctions [20]. Mitochondria are highly dynamic organelles that support cellular energy metabolism. They not only are ATP producers, but are also essential for phospholipid biogenesis, calcium homeostasis and apoptosis [21]. Mitochondria form an interconnected network intimately integrated with other cellular compartments. Therefore, it is not surprising that many inherited and acquired diseases, including neurodegenerative disorders, are associated with mitochondrial deregulation [22].

Several mitochondrial activities such as dynamics, transport, ATP production and mitochondrial quality control, all being of relevant importance for neuronal survival, are affected by mutated TDP-43 [23]. Wang and co-workers demonstrated, in different cellular models, that mitochondrial biogenesis is impaired by TDP-43 molecular defects. Indeed, TDP-43 G298S and A382T localize more in mitochondria leading to reduced translation of the complex I (CI) subunits NADH dehydrogenase 3 (ND3) and ND6, therefore decreasing their levels and affecting ATP synthesis [24]. Reduced mitochondrial ATP synthesis due to suppressed CI activities, reduced mitochondrial membrane potential (MMP), and increased ROS (reactive oxygen species) determine the alterations of mitochondria observed in patients with TDP-43 proteinopathy [25]. Moreover, in vitro and in vivo models showed that mitochondria are fragmented when TDP-43 is mutated [24,26,27,28,29,30].

Taken together, all these studies demonstrate that ALS is characterized by metabolic dysfunction, providing evidence that ALS pathogenesis is related to mitochondria pathology. Even though some of the most common ALS-related pathogenetic substitutions have been quite well characterized, little is known about TDP-43^G376D^. Here, we show that the G376D mutated protein is more localized to mitochondria, negatively affecting mitochondrial functionality. Indeed, we demonstrated that TDP-43^G376D^ impacts the respiratory chain CI and II, mitochondrial dynamics, increasing mitochondrial fragmentation, and that it is associated with increased oxidative stress.

## 2. Materials and Methods

### 2.1. Antibodies

Primary antibodies used in this study were mouse monoclonal anti-GFP (1:500, sc-9996), goat monoclonal anti-Lamin B (1:250, sc-6216), and mouse monoclonal anti-COX2 (1:200, sc-514489) from Santa Cruz Biotechnologies (Santa Cruz, CA, USA); mouse monoclonal anti-tubulin (1:10,000, T5168) and mouse monoclonal anti-Catalase antibody (1:1000, C0979) from Sigma-Aldrich (St. Louis, MO, USA); rabbit monoclonal anti-TOM20 antibody (1:1000, ab186734), Total OXPHOS Rodent Antibody Cocktail (1:500, ab110413), Complex II antibody cocktail (1:500, ab110410), rabbit monoclonal anti-SOD-2 (1:1000, ab68155), and rabbit monoclonal anti-GPX4 (1:1000, ab125066) from Abcam (Cambridge, UK); rabbit polyclonal anti-GPX1 (1:1000, GTX03346) from Genetex (Irvine, CA, USA); rabbit polyclonal anti-PGC1α (1:1000, A12348), monoclonal rabbit anti-TFAM (1:6000, A3173), rabbit polyclonal anti-NRF1 (1:1000, A5547), rabbit polyclonal anti-DRP1 (1:8000, A2586), rabbit polyclonal anti-FIS1 (1:500, A21527), rabbit monoclonal anti-MFN2 (1:1000, A19678), rabbit polyclonal anti-OPA1 (1:7000, A9833), rabbit polyclonal anti-cGAS (1:1000, A20125), and rabbit polyclonal anti-STING (1:1000, A21051) from Abclonal (Düsseldorf, Germany). Secondary antibodies conjugated to horseradish peroxidase (HRP) were all from Biorad (Hercules, CA, USA).

### 2.2. Cell Lines

Dermal fibroblasts were derived from skin biopsies of two healthy male controls and two ALS male patients in the fully symptomatic stages and indicated as ALS1A and ALS2A (A = advanced). ALS1O cells were derived from patient 1 at the early stages of the disease (O = onset). Patient 1 was 38 and 42 years old when the two skin biopsies were performed, from which the ALS1O and ALS1A fibroblasts were obtained. Fibroblasts were cultured as previously described [31]. HEK293T and Neuro2a cell lines were obtained from the ATCC and cultured in DMEM supplemented with 10% FBS, 2 mM L-glutamine, 100 U/mL penicillin, and 10 mg/mL streptomycin. All reagents for cell culture were purchased from Euroclone (Pero, MI, Italy). Cells were regularly evaluated for *Mycoplasma* contamination.

### 2.3. Plasmids and Transfection

The plasmids used in this study have been previously described [19]. HEK293T and Neuro2a cells were transfected as previously described using Metafectene Pro (Biontex, Munich, Germany) using the manufacturer’s instructions [32]. Cells were analyzed 48 h after transfection.

### 2.4. Measurement of Hydrogen Peroxide (H_2_O_2_)

H_2_O_2_ levels were measured in HEK293T and Neuro2a cells using a commercial kit (Abcam, ab102500) as described by Senese et al. [33]. In total, 2 × 10^6^ cells were resuspended in assay buffer and centrifuged at 14,000× *g* for 2–5 min at 4 °C. A deproteinization protocol was performed using 4 M perchloric acid (PCA), which was neutralized with ice-cold 2 M KOH, and the pH was adjusted to 6.5–8. The sample was then centrifuged at 13,000× *g* for 15 min at 4 °C. Subsequently, a reaction mix was prepared, and 50 µL of both the mix and the sample were added to each well. After 10 min of incubation, fluorescence was measured using a BioTek Synergy H1 microplate reader (Ex/Em = 535/587 nm) (Agilent, Santa Clara, CA, USA).

### 2.5. Western Blotting

Cell lysis was performed in a Laemmli buffer or RIPA (Radioimmunoprecipitation Assay) buffer, the compositions of which have been previously described [34]. Lysates were subjected to SDS-PAGE, and proteins were then transferred onto a PVDF (polyvinylidene fluoride) membrane from Millipore (Billerica, MA, USA). Membranes have been blocked in 5% milk in PBS (phosphate-buffered saline) and probed with primary and HRP-conjugated secondary antibodies diluted in 1% milk in PBS. Bands were visualized following incubation of membranes with Clarity or Clarity Max from Biorad (Hercules, CA, USA) using a Chemidoc MP Imaging System (Biorad). Bands were analyzed using ImageLab software 6.1 (Biorad, Hercules, CA, USA).

### 2.6. Seahorse XF Flux Assay

Mitochondrial activity and oxygen consumption rate (OCR) were evaluated with the Mito Stress Test Kit, while ATP production was measured with the Real-time ATP Rate Assay, both from Agilent Technology (Santa Clara, CA, USA). An 8-well Seahorse XFp Cell Culture Miniplate was used for transfected Neuro2a seeding at a density of 1.2 × 10^4^ cells/well in DMEM complete medium, and cells were cultivated at 37 °C in an incubator with 5% CO_2_. The following day, in order to induce differentiation, two washes with PBS were performed and cells were cultivated with DMEM without FBS for 24 h. The next day, the medium was changed with Seahorse XF DMEM medium supplemented as previously described [35]. Cells were incubated for 1 h at 37 °C without CO_2_. The Mito Stress Test and the Real-time ATP Rate Assay were performed as previously described [35].

### 2.7. Oxygen Consumption of Intact Cells

Oxygen consumption by HEK293T and Neuro2a cells was measured using an Oxygraph-2K respirometer (Oroboros, Innsbruck, Austria). Following trypsinization, the cells were resuspended in DMEM with low glucose (5 mM). The cells were centrifuged for 5 min at 350× *g* at room temperature. The cell pellet was then resuspended in an appropriate volume of DMEM low glucose to reach a final density of 1 × 10^6^ cells/mL. After instrument calibration, the respiration medium was aspirated from the oxygraph chamber, and 2 mL of cell suspension was added. Oxygen consumption was measured under controlled conditions (37 °C and stirring at 300 rpm). The respiratory states were assessed as follows: Routine respiration was measured without substrate addition; Oligomycin-induced respiration was assessed after ATP synthase inhibition with oligomycin (2.5 µM); and maximal respiration was determined in the presence of the mitochondrial uncoupler carbonyl cyanide-4-(trifluoromethoxy)phenylhydrazone (FCCP, 1 µM).

### 2.8. Mitochondria Isolation and Determination of Cytochrome Oxidase Activity (COX)

COX activity was measured in mitochondria isolated from HEK293T and Neuro2a cells. Cell pellets were resuspended in an isolation medium and homogenized, followed by centrifugation to obtain the mitochondrial pellet. The pellet was washed, resuspended, and kept on ice. COX activity was measured as previously described [36]. Mitochondrial aliquots were incubated with 1.0 mg/mL Lubrol for 30 min at 0 °C and then resuspended in 2 mL of reaction medium containing 30 μM Cytochrome C, 4 μM rotenone, 0.5 mM dinitrophenol, 10 mM Na-malonate, and 75 mM HEPES (pH 7.4). After approximately 10 min, the addition of the substrate (4 mM Na ascorbate with 0.3 mM N,N,N′,N′-tetramethyl-p-phenylenediamine) and the measure of the oxygen consumption were carried out. Auto-oxidation of the substrate was monitored in parallel experiments without the mitochondrial homogenates. The protein content of the samples was quantified using the Bio-Rad DC method (Bio-Rad, Hercules, CA, USA).

### 2.9. Mitochondrial Membrane Potential (MMP) Assay

The MMP assay in HEK293T, Neuro2a, and dermal Fibroblasts was detected by a TMRE-Mitochondrial Membrane Potential Assay kit (ab113852; Abcam, Cambridge, UK) [37]. For the assay, 1 × 10^4^ cells were plated. A fluorescence microscope (Leica DMLB; Leica Microsystems, Wetzlar, Germany) was used to visualize TMRE-positive cells.

### 2.10. Live Microscopy

Cells were seeded into 8-well µ-slides (Ibidi GmBh, Martinsried, Germany), and the next day, they were incubated with MitoTracker Red CM-H_2_XROS (50 nM) from ThermoFisher Scientific (Waltham, MA, USA) for 45 min at 37 °C in an incubator with 5% CO_2_ in DMEM without FBS. Three washes with PBS were performed, and L-15 medium (Leibowitz medium without phenol red, Gibco, ThermoFisher) was added to cells, then visualized using a LSM 700 confocal laser scanning microscope (Zeiss, Jena, Germany). ZEN Black Edition 2011 software (Zeiss) was used for image acquisition and ImageJ software (Version 1.5Oi, Bethesda, MD, USA) for fluorescence intensity quantification.

### 2.11. Statistical Analysis

Corrected Total Cell Fluorescence (CTCF) was calculated to quantify fluorescence intensity, as previously described [31]. As previously described, the number of individuals, networks, branches, and mitochondrial footprints was obtained using ImageJ software [38]. For all the analysis, at least 50 cells/samples of three independent experiments were analyzed. Data represents the mean ± standard error mean (SEM). Student’s *t*-test or one-way ANOVA followed by Tukey’s multiple comparison tests (* = *p*  ≤  0.05, ** = *p*  ≤  0.01, and *** = *p*  ≤  0.001) were used for statistical analysis.

## 3. Results

### 3.1. TDP-43^G376D^ Shows Higher Mitochondrial Localization and Impairs Complex I and Complex II of the Respiratory Chain

**Figure 1:**
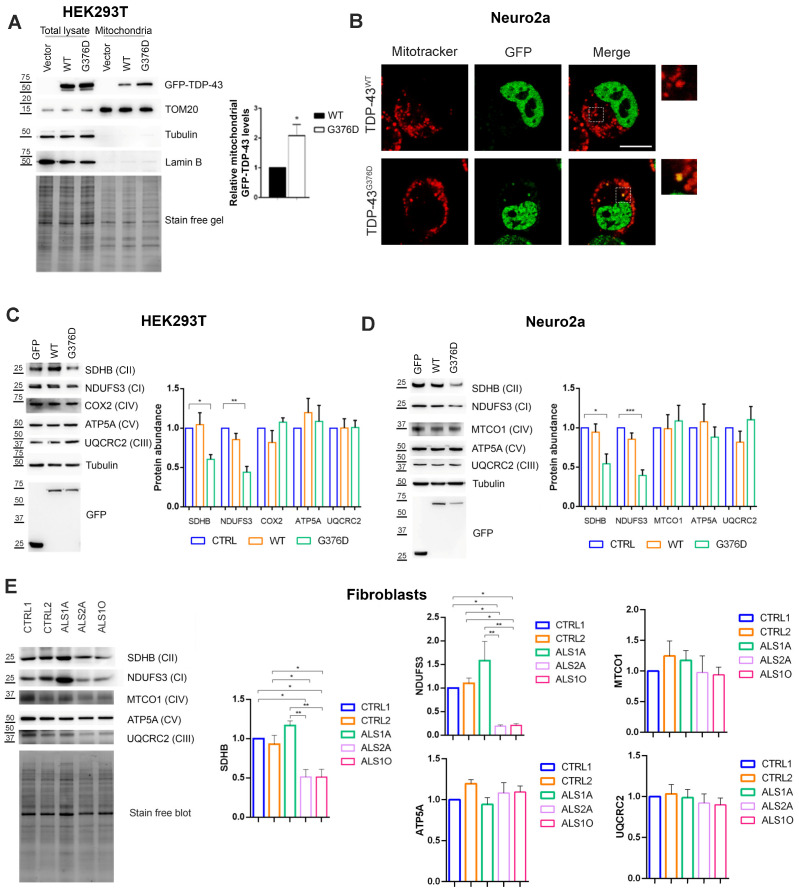
TDP-43^G376D^ localizes more in mitochondria and affects complex I and complex II of the respiratory chain. (**A**) HEK293T cells were transfected with pEGFPC1, pEGFPC1-TDP-43 WT or G376D vectors. Cells were lysed 48 h after transfection, and mitochondria were isolated by differential centrifugation. Samples were subjected to western blot analysis using antibodies against TDP-43, TOM20 (Mitochondrial import receptor subunit), Tubulin, and Lamin B. Total proteins were used as loading controls. Graphs represent the mean of at least three independent experiments ± s.e.m. Statistical analysis was performed using Student’s *t*-test. * = *p* < 0.05. (**B**) Neuro2a cells were transfected with pEGFPC1, pEGFPC1-TDP-43 WT or G376D vectors. cells were seeded into IBIDI microscopy chambers 24 h after transfection, and the next day, they were treated with 500 nM Mitotracker Red CM-H2XROS for 45 min in DMEM without serum at 37 °C. After three washes in PBS, Leibovitz’s medium without phenol red was added, and cells were imaged live by confocal microscopy. Bar = 10 µm. Inserts on the right represent magnification of the white dashed boxes. (**C**,**D**) HEK293T and Neuro2a cells were transfected with pEGFPC1, pEGFPC1-TDP-43 WT or G376D vectors. cells were lysed 48 h after transfection, and samples were subjected to western blot analysis using antibodies against SDHB, NDUFS3, COX2, MTCOI, ATP5A, UQCRC2, GFP and Tubulin, as loading control. (**E**) Fibroblasts were lysed, and samples were subjected to western blot analysis using antibodies against SDHB, NDUFS3, MTCOI, ATP5A and UQCRC2. Stain-free technology was used for loading control. (**C**–**E**) Graphs show the mean of at least three independent experiments ± s.e.m. Statistical analysis was performed using one-way ANOVA followed by Tukey’s test for multiple comparisons * = *p* < 0.05; ** = *p* < 0.01; *** = *p* < 0.001.

To evaluate if the G376D pathogenetic substitution in TDP-43 induces its mitochondrial localization, we transfected HEK293T cells with plasmids encoding GFP, GFP-TDP-43 wild-type or G376D, purified mitochondria by differential centrifugation, and evaluated the abundance of the mutated protein in mitochondria compared to wild-type protein. The mutated TDP-43 protein was more abundant in mitochondria compared to the wild-type counterpart (Figure 1A).

Then, we used another cellular model, Neuro2a cells, that were treated with Mitotracker Red after 48 h of transfection in order to visualize mitochondria and evaluate TDP-43 and mitochondria co-localization. While wild-type TDP-43 was localized in the nucleus, mutated TDP-43 showed also cytoplasmic localization, and some non-nuclear TDP-43 co-localized with mitochondria (Figure 1B), confirming the mitochondrial localization of the mutant protein.

Considering that it has been previously demonstrated that other ALS-associated pathogenetic substitutions caused increased TDP-43 mitochondrial localization affecting the expression of respiratory complex I (CI) [24], we wondered if TDP-43^G376D^ could also impair complexes of mitochondrial electron respiratory chain (ETC). Therefore, we evaluated the abundance of mitochondrial protein subunit of ETC in transfected HEK293T and Neuro2a cells, demonstrating that TDP-43^G376D^ affected NDUFS3 (NADH:Ubiquinone Oxidoreductase Core Subunit S3) (belonging to CI) and SDHB (Succinate Dehydrogenase B) (belonging to CII) expression in both cellular models (Figure 1C,D). Having demonstrated that TDP-43^G376D^ negatively influences NDUFS3 and SDHB expression, we analyzed the expression of the same proteins in control and ALS fibroblasts carrying the G376D pathogenetic substitution. Interestingly, ALS2A and ALS1O cells showed reduced expression of both mitochondrial subunits of CI and CII compared to control cells while ALS1A cells had increased expression of both NDUFS3 and SDHB (Figure 1E).

These data demonstrate that the increased mitochondrial localization of TDP-43^G376D^ has a negative impact on CI and II of the mitochondrial respiratory chain.

### 3.2. TDP-43^G376D^ Deeply Affects Mitochondrial Respiration

**Figure 2:**
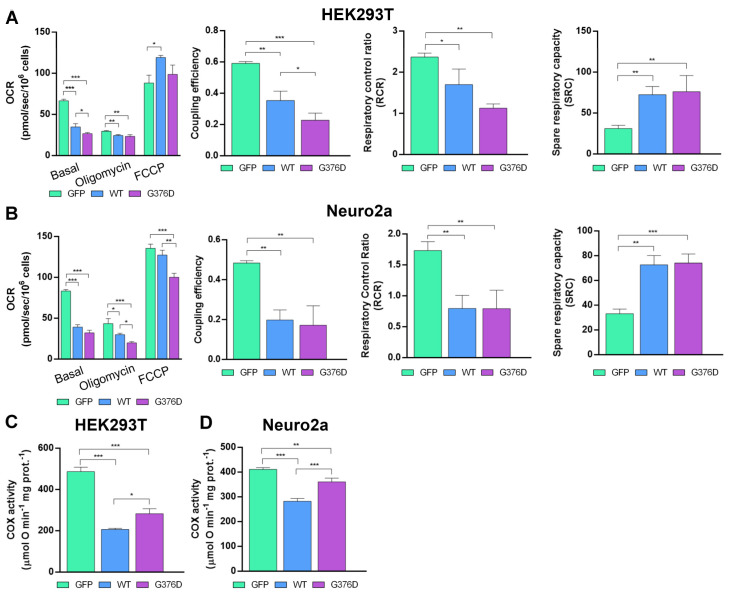
(**A**,**B**) Oxygen consumption rate (OCR) in HEK293T and Neuro2a cells measured with the Oroboros O2k system. Histograms represent basal respiration, oligomycin-sensitive respiration (indicating ATP-linked oxygen consumption), and maximal respiration after FCCP addition (reflecting mitochondrial maximal respiratory capacity). (**C**,**D**) The activity of cytochrome oxidase in mitochondria isolated from HEK293T and Neuro2a cells. All graphs represent the mean  ±  SEM of at least three independent experiments. Statistical analysis was performed using one-way ANOVA followed by Tukey’s test for multiple comparisons. * = *p* < 0.05; ** = *p* < 0.01; *** = *p* < 0.001.

To evaluate whether TDP-43^G376D^, significantly influencing CI and CII subunit expression, can affect mitochondrial respiration, we transfected HEK293T and Neuro2a cells with plasmids encoding GFP, GFP-TDP-43 wild-type, or GFP-TDP-43 G376D for 48 h. Mitochondrial respiration was then assessed in the presence of DMEM with low glucose. Figure 2 shows the results of experiments performed to determine the oxygen consumption rate (OCR) in suspended intact cells. Our results revealed that, in HEK293T cells overexpressing both wild-type and mutated TDP-43, basal respiration, leak respiration, coupling efficiency, and the respiratory control ratio (RCR) were all decreased compared to the GFP control. However, the decrease was more pronounced in the TDP-43^G376D^ cells compared to the wild-type TDP-43 cells (Figure 2A). Maximal uncoupled respiration (FCCP respiration) was increased in cells overexpressing wild-type TDP-43 compared to the GFP control (Figure 2A).

The spare respiratory capacity was elevated in both cells expressing TDP-43^G376D^ and those overexpressing wild-type TDP-43 (Figure 2A). In Neuro2a cells, basal respiration, leak respiration, FCCP respiration, coupling efficiency, and RCR were all decreased in both TDP-43^G376D^ and wild-type TDP-43 cells compared to the GFP control. Also, in Neuro2a cells, the decrease was more pronounced in the TDP-43^G376D^ cells compared to the wild-type TDP-43 cells (Figure 2B). However, spare respiratory capacity was increased in both cells expressing TDP-43^G376D^ and those overexpressing wild-type TDP-43 compared to the GFP control (Figure 2B). To obtain a more comprehensive understanding of mitochondrial respiration, we measured the Cytochrome C Oxidase (COX) activity. The results showed a significant reduction in COX activity in HEK293T and Neuro2a cells expressing TDP-43^G376D^, as well as in cells overexpressing wild-type TDP-43, compared to the GFP control (Figure 2C,D).

**Figure 3:**
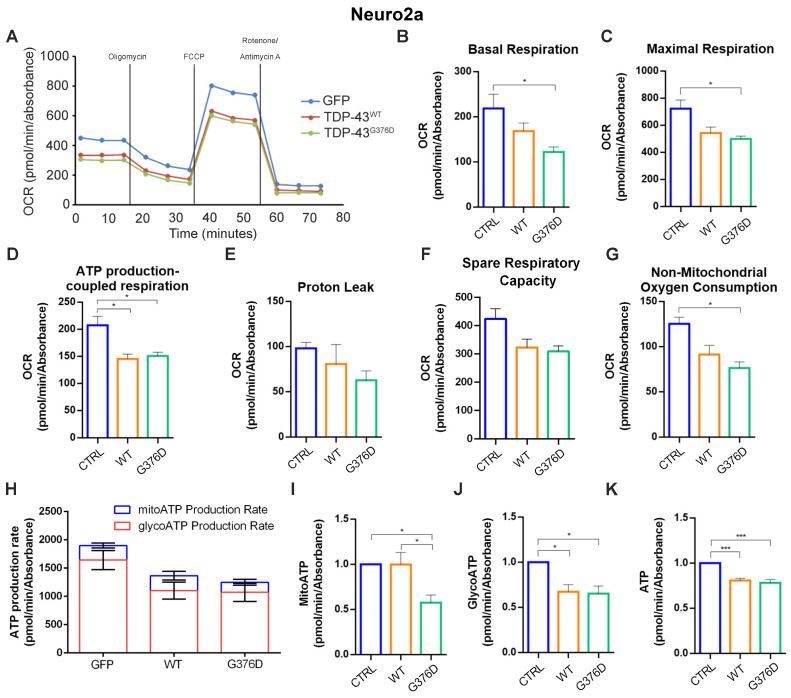
TDP-43^G376D^ affects mitochondrial functionality. Transfected Neuro2a cells were differentiated by removing serum from the medium for 24 h. (**A**) Oxygen consumption rate (OCR) was determined with the Seahorse Mito stress kit assay. (**B**–**G**) Basal respiration, maximal respiration, ATP-production coupled respiration, proton leak, spare respiration capacity, and non-mitochondrial oxygen consumption were determined by Seahorse data elaboration. (**H**) ATP production rate was measured by the Seahorse instrument during a Real-Time ATP Rate assay, and (**I**–**K**) subsequent elaboration data have allowed determining ATP produced by mitochondria (MitoATP), by the glycolysis route (GlycoATP), and total ATP. All graphs represent the mean  ±  SEM of at least three independent experiments. Statistical analysis was performed using one-way ANOVA followed by Tukey’s test for multiple comparisons. * = *p*  <  0.05; *** = *p*  <  0.001.

To further confirm the alteration of mitochondrial functions, we used the Seahorse Mito Stress Kit assay in transfected and differentiated Neuro2a cells. We found that in cells overexpressing wild-type and mutated TDP-43, the OCR was decreased compared to GFP-expressing cells, used as the control (Figure 3A).

In addition, other parameters such as basal and maximal respiration, ATP production, proton leak, spare respiratory capacity, and non-mitochondrial oxygen consumption were downregulated in cells overexpressing wild-type or mutated TDP-43, but the latter had the worst effects (Figure 3B–G). Moreover, when we analyzed ATP production using the Real-Time ATP Rate assay, we found a significant reduction in ATP levels, both mitochondrial and glycolytic, in cells expressing TDP-43^G376D^, while we found only a reduction in glycolytic ATP in cells overexpressing wild-type TDP-43 (Figure 3H–K).

**Figure 4:**
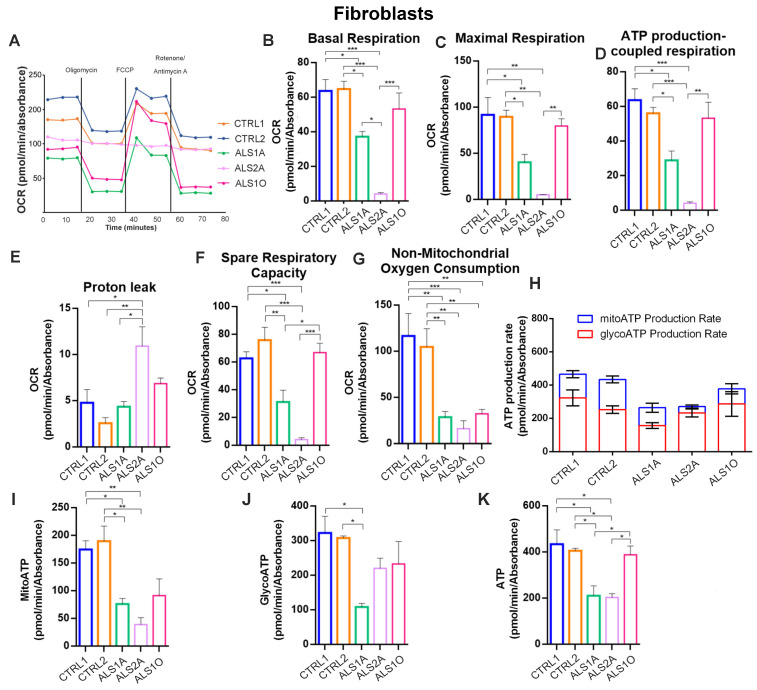
Mitochondrial functionality was compromised in ALS fibroblasts. (**A**) Oxygen rate consumption (OCR) was determined in control and ALS fibroblasts with the Seahorse Mito stress kit assay. (**B**–**G**) Basal respiration, maximal respiration, ATP-production coupled respiration, proton leak, spare respiration capacity, and non-mitochondrial oxygen consumption were determined by Seahorse data elaboration. (**H**) The ATP production rate was measured by a Seahorse instrument during the Real-Time ATP Rate assay, and (**I**–**K**) subsequent elaboration data have allowed determining ATP produced by mitochondria (MitoATP), by the glycolysis route (GlycoATP), and total ATP. All graphs represent the mean  ±  SEM of at least three independent experiments. Statistical analysis was performed using one-way ANOVA followed by Tukey’s test for multiple comparisons. * = *p*  <  0.05; ** = *p*  <  0.01; *** = *p*  <  0.001.

The Seahorse Mito Stress Kit assay showed alterations in the functionality of mitochondria also in fibroblasts. Indeed, we observed a decrease in the OCR, and this was particularly strong in ALS2A cells (Figure 4A).

Furthermore, all the other parameters, such as basal and maximal respiration, ATP production, spare respiratory capacity, and non-mitochondrial oxygen consumption, except for proton leak, were significantly decreased in ALS cells (Figure 4B–G). The Real-Time ATP Rate assay confirmed the data obtained in transfected cells with a reduction of ATP production in ALS cells, both glycolytic and mitochondrial (Figure 4H–K). Interestingly, all these parameters in ALS1O cells were more similar to control cells, suggesting that mitochondrial functionality could become worse with the progression of the disease.

Altogether, these data indicate that mitochondrial functionality is deeply compromised by TDP-43^G376D^.

### 3.3. The G376D Pathogenetic Substitution in TDP-43 Causes Mitochondrial Fragmentation and the Reduction of the MMP

**Figure 5:**
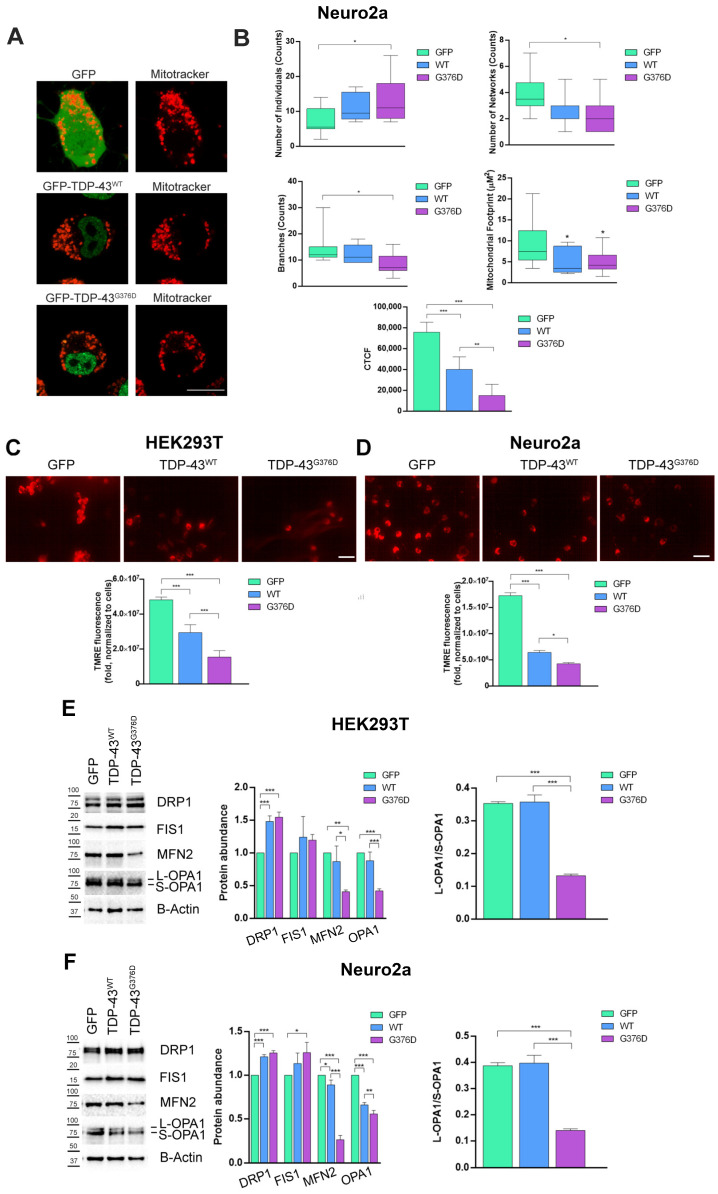
TDP-43^G376D^ determines mitochondrial fragmentation and reduces MMP. (**A**) Neuro2a cells were transfected with pEGFPC1, pEGFPC1-TDP-43 WT or G376D vectors. Cells were seeded into IBIDI microscopy chambers 24 h after transfection, and the next day, they were treated with 500 nM Mitotracker Red CM-H2XROS for 45 min in DMEM without serum at 37 °C. After three washes in PBS, Leibovitz’s medium without phenol red was added, and cells were imaged live by confocal microscopy. Bar = 10 µm (**B**) The number of individuals, networks, branches, mitochondrial footprint, and CTCF were calculated using the ImageJ software. (**C**,**D**) Determination of mitochondrial membrane potential in HEK293T and Neuro2a cells through detecting TMRE-positive cells (red). The images were captured at ×40 magnification under a fluorescence microscope. Scale bars represent 40 µm. The histogram shows TMRE fluorescence intensity that was analyzed by ImageJ software. (**E**,**F**) HEK293T and Neuro2a cells were transfected with pEGFPC1, pEGFPC1-TDP-43 WT or G376D vectors. Cells were lysed 48 h after transfection, and samples were subjected to western blot analysis using antibodies against DRP1, FIS1, MFN2, OPA1, and B-ACTIN as loading control. The quantification of the ratio between L-OPA1 and S-OPA1 was shown. All graphs represent the mean ± SEM of at least three independent experiments. Statistical analysis was performed using one-way ANOVA followed by Tukey’s test for multiple comparisons. * = *p*  <  0.05; ** = *p*  <  0.01; *** = *p*  <  0.001.

Alterations in mitochondrial dynamics and bioenergetics defects are closely linked [39]; thus, to understand if the TDP-43 causative variant can affect mitochondrial morphology and networking, we transfected Neuro2a cells with plasmids coding for GFP, GFP-TDP-43 wild-type or G376D, and we stained organelles with Mitotracker Red CM-H_2_XROS (Figure 5A).

Then, we performed a mitochondrial network analysis which demonstrated an increased number of individuals and the reduction of the mitochondrial network and branches (Figure 5B). These data highlighted the fragmentation of the mitochondrial network. Moreover, we demonstrated a decreased mitochondrial mass, as indicated by the reduced mitochondrial footprint in TDP-43^G376D^-expressing cells, and a compromised MMP, as shown by the reduction of CTCF (Figure 5B). Indeed, although MitoTracker Red CM-H2XROS is not a potentiometric dye, its accumulation in cells depends on MMP and intensity of fluorescence can be used as an indirect measure of MMP alterations [40]. Decreased mitochondrial mass and mitochondrial CTCF were also observed in cells overexpressing TDP-43 wild-type (Figure 5A,B).

Reduced MMP in TDP-43^G376D^-expressing cells was also confirmed by fluorescence microscopy using TMRE mitochondrial staining in transfected HEK293T and Neuro2a cells (Figure 5C,D). Our data showed a significant decrease in MMP in both cells expressing TDP-43^G376D^ and those overexpressing wild-type TDP-43 compared to GFP expressing cells. However, this decrease was more pronounced in the TDP-43^G376D^ cells compared to the wild-type TDP-43 cells.

Considering the alteration of the mitochondrial network that we observed, we evaluated the abundance of dynamin-related protein 1 (DRP1) and mitochondrial fission 1 protein (FIS1), required for mitochondrial fission, and mitofusin 1 (MFN2) and optic atrophy 1 (OPA1), required for mitochondrial fusion [41]. As expected, Drp1 and FIS1 abundance were increased in HEK293T and Neuro2a cells overexpressing wild-type and mutated TDP-43, while the abundance of proteins important for mitochondrial fusion was reduced (Figure 5E,F). Moreover, we also observed a reduced ratio between L-OPA1 (long OPA1) and S-OPA1 (short OPA1), further corroborating the observed mitochondrial fragmentation (Figure 5E,F).

These results indicate that cells overexpressing wild-type TDP-43, as well as those carrying the TDP-43^G376D^ causative variant, exhibit alterations in mitochondrial dynamics. The increase in mitochondrial fission and the decrease in mitochondrial fusion contribute to mitochondrial fragmentation, dysfunction, and energy deficits.

**Figure 6:**
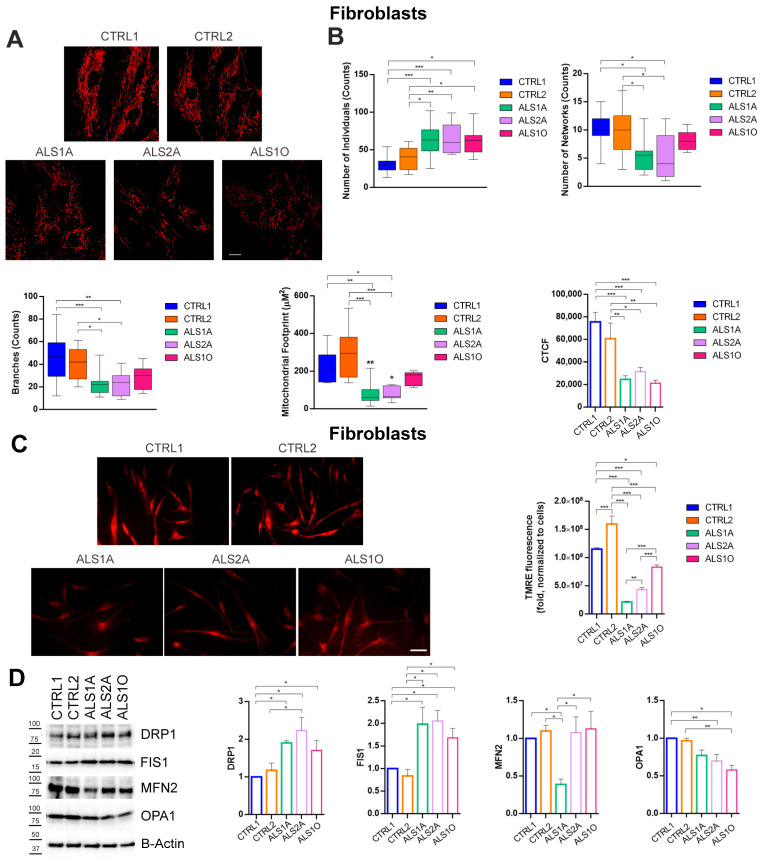
The mitochondrial network is fragmented in ALS fibroblasts. (**A**) Control and ALS fibroblasts were seeded into IBIDI microscopy chambers, and the next day, they were treated with 500 nM Mitotracker Red CM-H2XROS for 45 min in DMEM without serum at 37 °C. After three washes in PBS, Leibovitz’s medium without phenol red was added and cells were live imaged by confocal microscopy. Bar = 10 µm (**B**) The number of individuals, networks, branches, mitochondrial footprint, and CTCF were calculated using the ImageJ software. (**C**) Determination of mitochondrial membrane potential in control and ALS fibroblast cells through detecting TMRE-positive cells (red). The images were captured at ×40 magnification under a fluorescence microscope. Scale bars represent 40 µm. The histogram shows TMRE fluorescence intensity that was analyzed by ImageJ software. (**D**) Control and ALS fibroblasts were lysed, and samples were subjected to western blot analysis using antibodies against DRP1, FIS1, MFN2, OPA1, and B-ACTIN as a loading control. All graphs show the mean of at least three independent experiments ± s.e.m. Statistical analysis was performed using one-way ANOVA followed by Tukey’s test for multiple comparisons. *** = *p* < 0.001; ** = *p* < 0.01; * = *p*  <  0.05.

ALS fibroblasts also showed mitochondrial fragmentation with an increase in the number of individuals in all patients’ cells and a decrease in the number of networks and branches in the two patient cells with advanced disease (Figure 6A,B). Also, reduced mitochondrial mass and mitochondrial fluorescent intensity (CTCF) were found in patient cells compared to control cells (Figure 6A,B). Moreover, our data showed a significant decrease in MMP in ALS fibroblasts carrying the G376D causative variant compared to the control. However, the decrease is particularly pronounced in ALS1A and ALS2A cells, suggesting that it could become worse with disease progression (Figure 6C).

Regarding mitochondrial dynamics in dermal fibroblasts, it was observed that the protein levels of DRP1 and FIS1 were significantly increased in fibroblasts from ALS patients carrying the G376D pathogenetic substitution, compared to controls. Additionally, the abundance of the protein OPA1 was reduced in ALS fibroblasts compared to the control group, suggesting an alteration in mitochondrial fusion. At the same time, the abundance of MFN2 was drastically decreased in ALS fibroblasts, particularly in the advanced stages of the disease, indicating a possible involvement of this protein in the pathological mechanisms underlying ALS progression (Figure 6D).

Altogether, these data demonstrate that TDP-43^G376D^ determines mitochondrial fragmentation and negatively affects MMP.

### 3.4. The G376D Pathogenetic Substitution in TDP-43 Affects Mitochondrial Biogenesis and Is Related to Increased Oxidative Stress

**Figure 7:**
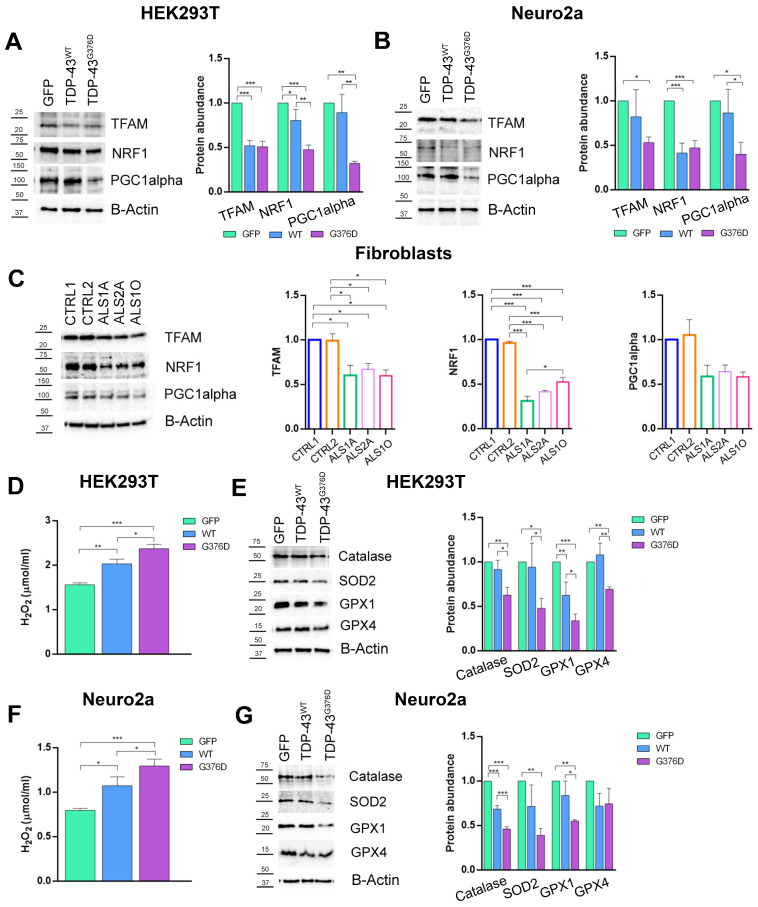
TDP-43^G376D^ decreases mitochondrial biogenesis and increases oxidative stress. (**A**,**B**) HEK293T and Neuro2a cells were transfected with pEGFPC1, pEGFPC1-TDP-43 WT or G376D vectors. Cells were lysed 48 h after transfection, and samples were subjected to western blot analysis using antibodies against PGC1α, NRF1, TFAM, and B-ACTIN as loading control. (**C**) Control and ALS fibroblasts were lysed, and samples were subjected to western blot analysis using antibodies against PGC1α, NRF1, TFAM, and B-ACTIN as loading control. (**D**,**F**) Detection of H_2_O_2_ concentrations (µmol/mL) in HEK293T and Neuro-2a cells. (**E**–**G**) HEK293T and Neuro2a cells were transfected with pEGFPC1, pEGFPC1-TDP-43 WT or G376D vectors. Cells were lysed 48 h after transfection, and samples were subjected to western blot analysis using antibodies against Catalase, SOD-2, GPX-1, GPX-4, and B-ACTIN as the loading control. All graphs represent the mean ± SEM of at least three independent experiments. Statistical analysis was performed using one-way ANOVA followed by Tukey’s test for multiple comparisons. *** = *p* < 0.001; ** = *p* < 0.01; * = *p* < 0.05.

Impaired mitochondrial biogenesis is a key feature in the pathophysiology of ALS, and alterations in this process can exacerbate mitochondrial dysfunction [42,43]. To this end, we evaluated the abundance of key regulators of mitochondrial biogenesis in HEK293T, Neuro2a, and dermal fibroblast cells, including Peroxisome Proliferator-Activated Receptor Gamma Coactivator 1-alpha (PGC1α), Nuclear Respiratory Factor 1 (NRF1), and Mitochondrial Transcription Factor A (TFAM). Our results showed that the abundance of TFAM, NRF1, and PGC1α was decreased in both HEK293T (Figure 7A) and Neuro2a (Figure 7B) cells expressing TDP-43^G376D^ and those overexpressing wild-type TDP-43, compared to GFP. However, the reduction was more pronounced in the TDP-43^G376D^ cells compared to the wild-type TDP-43 cells. The reduction in mitochondrial biogenesis was also observed in dermal fibroblasts. The protein levels of TFAM, NRF1, and PGC1α were reduced in ALS fibroblasts carrying the G376D causative variant compared to the control group (Figure 7C). This suggests an alteration in the regulatory pathways involved in mitochondrial biogenesis, which may contribute to the mitochondrial dysfunction observed in ALS.

Dysfunctional mitochondria produce more ROS, which can further damage mitochondria, reduce MMP, and cause oxidative damage to cells. Our results showed a significant increase in H_2_O_2_ levels in both HEK293T (Figure 7D) and Neuro2a (Figure 7F) cells expressing TDP-43^G376D^, as well as in cells overexpressing wild-type TDP-43, when compared to the GFP control group. Interestingly, while both TDP-43^G376D^ and wild-type TDP-43 expressing cells exhibited elevated levels of H_2_O_2_, the increase in ROS production was notably more pronounced in the cells expressing TDP-43^G376D^, suggesting that the mutant form of the protein may induce a greater level of oxidative stress than the overexpression of wild-type protein.

Subsequently, we evaluated the antioxidant machinery using Western blot analysis. The protein levels of Catalase, Superoxide dismutase 2 (SOD-2), Glutathione peroxidase 1 (GPX1), and Glutathione peroxidase 4 (GPX4) were significantly decreased in both HEK293T (Figure 7E) and Neuro2a (Figure 7G) cells expressing TDP-43^G376D^, as well as in cells overexpressing wild-type TDP-43, compared to the GFP control group. The reduction in antioxidant protein levels was more pronounced in the cells expressing TDP-43^G376D^.

These findings collectively suggest that TDP-43^G376D^ induces a chronic oxidative stress condition, which is associated with an imbalance in the antioxidant defense system.

### 3.5. TDP-43^G376D^ Activates the cGAS-STING Pathway

**Figure 8:**
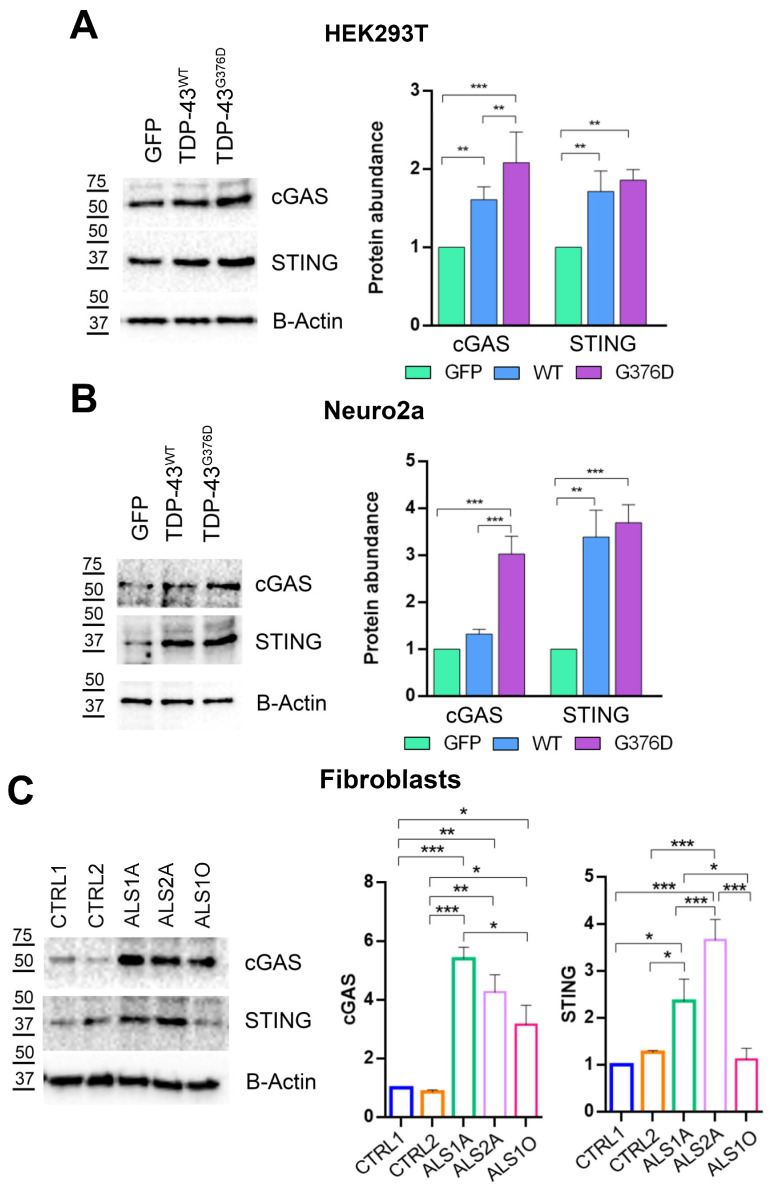
TDP-43G376D activates the cGAS-STING pathway. (**A**,**B**) HEK293T and Neuro2a cells were transfected with pEGFPC1, pEGFPC1-TDP-43 WT or G376D vectors. Cells were lysed 48 h after transfection, and samples were subjected to western blot analysis using antibodies against cGAS, STING and B-ACTIN as loading control. (**C**) Control and ALS fibroblasts were lysed, and samples were subjected to western blot analysis using antibodies against cGAS, STING and B-ACTIN as loading control. The graphs show the mean of at least three independent experiments ± s.e.m. Statistical analysis was performed using one-way ANOVA followed by Tukey’s test for multiple comparisons. *** = *p* < 0.001; ** = *p* < 0.01; *p < 0.05.

In order to evaluate if mitochondrial dysfunction could lead to neuroinflammation, the activation of the cGAS-STING pathway was evaluated by measuring the protein levels of these two markers. Our results showed a significant increase in cGAS protein levels in both HEK293T and Neuro2a cells expressing TDP-43^G376D^, as well as in those overexpressing wild-type TDP-43 compared to the control group (Figure 8A,B).

Furthermore, this increase was more pronounced in the TDP-43^G376D^ cells than in the wild-type TDP-43 cells. Regarding STING protein levels, they were significantly increased in HEK293T and Neuro2a cells expressing TDP-43^G376D^, as well as in those overexpressing wild-type TDP-43 compared to the control group. Moreover, our data show that cGAS and STING protein levels are significantly enhanced in ALS fibroblasts carrying the G376D mutation compared to the controls. Notably, this increase is more pronounced in ALS1A and ALS2A (Figure 8C).

## 4. Discussion

Among the known genetic factors contributing to approximately 40–55% of familial ALS cases, there is *TARDBP* [44,45], and a novel causative variant in the *TARDBP* gene, p.G376D, was recently discovered in Italian, Swiss, and Asian families [16,17,18]. We previously demonstrated that expression in Neuro2a cells of the TDP-43^G376D^ mutant protein leads to increased cytoplasmic aggregation of TDP-43, and that patient fibroblasts carrying this pathogenetic substitution show the same phenotype, highlighting the mislocalization of the mutated protein [19]. This characteristic resembles the pathological aggregates found in tissues derived from both familial and sporadic ALS patients, closely mimicking TDP-43 proteinopathy [46,47]. However, at present little is known about this new and rare causative variant.

As mitochondrial dysfunction contributes to motor neuron degeneration in ALS models [48,49,50,51,52], we investigated whether and how mitochondrial function might be altered in the presence of the TDP43^G376D^ mutant protein. Specifically, the analysis was carried out on HEK293T and Neuro2a cells transfected with wild-type or mutated TDP-43, and on dermal fibroblasts obtained from skin biopsies of healthy male controls and ALS male patients carrying TDP-43^G376D^. Although the patient cohort is tiny as a consequence of the rarity of this molecular defect, for patient 1, we analyzed fibroblasts obtained after skin biopsy at both the early and advanced stages of the disease. This is an original peculiarity of our work.

We determined that the TDP-43^G376D^ protein is more localized to mitochondria compared to the wild-type counterpart (Figure 1A,B), similar to the previously characterized G298S, A315T, and A382T pathogenetic substitutions [24]. It was proposed that TDP-43 mitochondrial localization results in the downregulation of respiratory chain subunits, specifically of CI [24]. In addition, the expression of TDP-43^G376D^ in both transfected cell lines strongly reduced the abundance of NDUFS3 and SDHB, which are subunits of mitochondrial respiratory CI and II, respectively (Figure 1C,D). A similar effect was detected in ALS2A and ALS1O fibroblasts compared to control cells (Figure 1E). The decrease in the abundance of these complexes can lead to impaired ATP production and increased oxidative stress. On the other hand, it was previously described that ALS-associated TDP-43 causative variants (A382T and G298S) led to a decrease in OCR and ATP production, with a less severe phenotype associated with the overexpression of the wild-type protein compared to the expression of the mutant protein [24]. To better understand if the downregulation of CI and CII subunits induced by TDP-43^G376D^ was associated with alterations in mitochondrial function, we analyzed OCR with two systematic approaches. In the first instance, we evaluated using high-resolution Oxygraph-2K respirometer, and in low glucose stress condition, OCR under basal conditions, when oxygen consumption was inhibited by the ATP synthesis blocker oligomycin, and when oxygen consumption was stimulated by the OXPHOS uncoupler FCCP. Our results indicated a reduction in OCR during baseline cellular respiration, after blocking ATP synthesis, and after uncoupling respiration in cells overexpressing wild-type and mutant TDP-43. However, the decrease was more pronounced in cells overexpressing TDP-43^G376D^ (Figure 2A,B). Furthermore, a decrease in COX activity was found upon overexpression of wild-type TDP-43 or of TDP-43^G376D^ (Figure 2C,D). As a second approach, we used Seahorse, and we observed that cells expressing TDP-43^G376D^ are characterized by a bioenergetic mitochondrial deficit leading to decreased basal and maximal mitochondrial respiration and a significant reduction of ATP production coupling respiration (Figure 3). Notably, we found that a reduction in total and glycolytic ATP production occurred also in cells overexpressing TDP-43^wt^, but the decrease in mitochondrial ATP production was a prerogative of the TDP-43 causative variant. This suggests the impossibility of compensating mitochondrial dysfunction through glycolytic ATP production. In this respect, ALS1O fibroblasts were more similar to control cells and showed a less severe phenotype, suggesting that mitochondrial dysfunctions worsen with the progression of the disease. In fact, ALS1A, and ALS2A fibroblasts especially, showed more severe alterations in mitochondrial respiration and ATP production compared to ALS1O fibroblasts. Clearly, to establish that the worst phenotype is due to the worsening of the disease and not to other factors (such as the age of sampling or else), the cohort of patients at early and advanced stages of the disease should be increased in the future. Interestingly, SDHB and NDUFS3 proteins in ALS1A fibroblasts are more abundant compared to other fibroblasts from patients, probably as a vain attempt to counteract the dysfunctions observed. Moreover, we observed a less severe reduction in glycolytic ATP production in ALS2A as a guarantee of cellular survival, and this may be the basis of the worst phenotype observed in ALS2A fibroblasts, possibly contributing to the worst clinical characteristics observed in this patient [19].

Previous studies have shown that the expression of wild-type TDP-43 or mutated TDP-43 (Q331K and M337V) in NSC-34 motoneurons leads to a decrease in MMP [53]. Moreover, Wang and co-workers have shown that cells overexpressing ALS-causing pathogenetic substitutions G298S, A315T, or A382T, or ALS fibroblasts carrying the G298S or A382T causative variants, exhibit accumulation of mutant TDP-43 within mitochondria. The mutant protein aggregates preferentially bind to ND3 and ND6 subunits, leading to the disassembly of CI and a reduction in MMP [24]. A significant decrease in MMP in cells overexpressing wild-type TDP-43 was observed, but this decrease was more pronounced in cells expressing the G376D pathogenetic substitution (Figure 5C,D). MMP was also evaluated in fibroblasts, showing a significant decrease in ALS fibroblasts at the advanced stages of the disease (Figure 6C). This observed reduction in MMP was consistent with the decrease in mitochondrial respiration, reflecting a deeper level of mitochondrial dysfunction.

To complete the picture of mitochondrial dysfunction, the evaluation of ROS production naturally followed. Indeed, it is almost dogmatic that mitochondria act as both a source and a target of reactive oxygen species (ROS), and we have not ruled out that the decrease in MMP and the observed impairment of mitochondrial function could induce alterations in ROS production and their scavenger systems. In this regard, a large body of evidence suggests that both increased ROS levels and impaired antioxidant defense play significant roles in ALS [54]. Indeed, for instance, an increase in ROS production was observed in an in vitro model of upper motor neurons transfected with wild-type TDP-43 or TDP-43^A315T^ [55]. Thus, we assessed both the production of ROS and the antioxidant defense system. Our analysis revealed that cells overexpressing wild-type TDP-43 exhibit increased levels of oxidative stress, but the increase was even more pronounced in cells carrying the G376D causative variant (Figure 7D–F). Moreover, these cells exhibited a reduced ability to counteract ROS overproduction, as shown by a decrease in the protein levels of mitochondrial SOD-2 and other key enzymes involved in antioxidant defense systems such as Catalase, GPX-1, and GPX-4 (Figure 7E–G).

Mitochondrial dysfunction, dissipation of MMP, and an increase in ROS production are events that are often accompanied or preceded by mitochondrial fission [56]. Mitochondrial homeostasis is characterized by an equilibrated dynamic process of fusion and fission to ensure cellular energy production, help mitigate oxidative stress, and support the overall health of the cell [57]. Fragmented mitochondria, with damaged inner membrane structures, have been increasingly identified as early hallmark features in several neurodegenerative diseases, including ALS [23,58,59]. Our study revealed an increased number of individuals and a reduction in the mitochondrial network and branches in TDP-43^G376D^-expressing Neuro2a cells (Figure 5A,B). Similar results were obtained in ALS fibroblasts carrying the causative variant, but the decrease in the number of networks and branches, as well as the increase in the number of individuals, was more evident at advanced stages of the disease (Figure 6A,B). The nature of the observed mitochondrial network changes was investigated by assessing key markers of both mitochondrial fission and fusion processes. We observed an increase in mitochondrial fission proteins and a decrease in proteins involved in mitochondrial fusion, which was more pronounced when TDP-43^G376D^ was present (Figure 5E,F). In ALS fibroblasts carrying the G376D causative variant, we observed a higher abundance of DRP1 and FIS1, while the abundance of OPA1 and MFN2 decreased in the advanced stages of the disease (Figure 6D). The changes observed align with studies that consistently report altered levels of proteins important for mitochondrial fission and fusion like DRP1, FIS1, MFN1, and OPA1 [60,61]. Indeed, overexpression of wild-type TDP-43 leads to an increase in mitochondrial fission markers and a decrease in both fusion markers [26] and excessive mitochondrial fission, coupled with the degradation of the mitochondrial inner membrane structure, occurring in neuronal cells expressing TDP-43 mutants [28,62,63]. Our results suggest that the molecular defect leads to alterations in mitochondrial dynamics. Increased mitochondrial fission and decreased fusion contribute to mitochondrial fragmentation, dysfunction, and energy deficits. Several pathways regulate mitochondrial dynamics and biogenesis in cells [64]. The increased mitochondrial fission is further corroborated by the increased ratio between two OPA1 isoforms, L-OPA1 and S-OPA1, observed in our cellular models expressing TDP-43^G376D^. Indeed, in HEK293T and Neuro2a, the balance showed a trend towards the short isoform (Figure 5E,F). According to the literature, the balance between the long and short isoforms of OPA1 is crucial for the maintenance of mitochondrial homeostasis. S-OPA1 primarily mediates mitochondrial fission, a process that can be activated in response to cellular stress, mitochondrial damage, or specific energy demands, as it allows the separation of mitochondria for easier elimination of damaged or dysfunctional mitochondria [65]. Moreover, oxidative stress and alterations in mitochondrial membrane potential are associated with increased S-OPA1 levels, and we observed both these phenotypes in our cellular models carrying TDP-43^G376D^ [66,67].

Mitochondrial biogenesis is crucial for maintaining mitochondrial balance throughout the cell’s life cycle, ensuring that new mitochondrial components are produced to replace damaged ones and support the cell’s physiological needs [68,69]. Several studies reported changes in mitochondrial biogenesis markers in both ALS mouse models and patients. A decrease in mitochondrial biogenesis markers in the spinal cord and muscle tissue of both mice and patients with the SOD1^G93A^ causative variant was observed. This reduction suggests a disruption in mitochondrial maintenance processes in ALS, potentially contributing to the disease’s pathophysiology [70,71]. We observed a reduction in PGC1α, TFAM, and NRF1 protein levels in HEK293T and Neuro2a cells expressing TDP-43^G376D^, as well as in those overexpressing wild-type TDP-43. Additionally, the protein levels of these markers were significantly reduced in ALS fibroblasts carrying the G376D pathogenetic substitution (Figure 7A–C). The observed reduction in mitochondrial biogenesis contributes to the accumulation of damaged mitochondria, further exacerbating the alteration of mitochondrial homeostasis. In our experimental models, we observed not only a reduction in PGC1α, TFAM, and NRF1 protein levels, but also a downregulation of complexes I and II of the mitochondrial respiratory chain, highlighting an impairment in the complexes that play a key role in generating the proton motive force. Indeed, Complex I is the first and largest enzyme in the mitochondrial respiratory chain, playing a key role in generating the proton motive force that drives ATP synthesis. Complex II, on the other hand, is located at the junction of two critical pathways, the Krebs cycle and oxidative phosphorylation, both of which are essential for ATP production. It has been suggested that the function, regulation, and response of complexes I and II to pathophysiological stimuli are crucial for cellular bioenergetics and may contribute to the development of neurodegenerative disease [72]. In addition, the unchanged protein levels of complexes III and IV could be read as a failure effort from the cell to maintain mitochondrial function despite a compromised mitochondrial compartment. The failure of this potential compensative mechanism is further supported by a reduction in the activity of Cytochrome C oxidase (complex IV).

The accumulation of dysfunctional mitochondria in the cell triggers the activation of the cGAS-STING pathway. The activation of this pathway has been implicated in various neurodegenerative diseases, including Alzheimer’s disease (AD), Parkinson’s disease (PD), and amyotrophic lateral sclerosis (ALS) [73]. We reported the induction of this pathway in HEK293T and Neuro2a cells overexpressing wild-type and mutated TDP-43, and in ALS fibroblasts carrying the G376D mutation (Figure 8). Particularly, the activation of this pathway is more pronounced at advanced stages of the disease, potentially suggesting the establishment of a neuroinflammatory condition. Moreover, previous work has demonstrated that mitochondrial localization of TDP-43 induces cytosolic translocation of mtDNA, where it accumulates triggering neuroinflammation in ALS patient iPSC-derived motor neurons and spinal cord samples by activation of cGAS-STING pathway [74]. Thus, we do not exclude that mitochondrial accumulation of the p.G376D TDP-43 form can exacerbate the effects of TDP-43 and influence signal transduction in mitochondria as a consequence of mtDNA cytosolic accumulation.

In conclusion, our study allows us to increase our knowledge of the molecular mechanisms underlying ALS disease associated with TDP-43^G376D^. Indeed, although the effect of TDP-43 on mitochondria was already described, this is the first work that elucidated the toxic role of the TDP-43^G376D^ mutant protein as a consequence of its mitochondrial accumulation. We know that the generalizability of this study may be limited by the low number of samples available, as this is a rare causative variant. However, this study has been performed not only on skin fibroblasts derived from two ALS patients but also in cell lines expressing the TDP-43^G376D^ mutant protein, demonstrating that the observed phenotypes are directly linked to the expression of the mutant protein and do not arise from individual differences between patient and control cells. In any case, the number of patients in the future will be expanded to strengthen our results, with the aim of contributing to the identification of cellular and molecular mechanisms underlying neurodegeneration, which could open the possibility of developing targeted therapy.

## 5. Conclusions

Our data indicate that TDP-43^G376D^ is more localized to mitochondria compared to the wild-type counterpart. This has severe consequences affecting complexes I and II of the respiratory chain and mitochondrial respiration. Moreover, upon expression of the TDP-43^G376D^, the mitochondrial dynamic shifted towards fragmentation, mitochondrial biogenesis was impaired, and oxidative stress was increased due to the reduced abundance of antioxidant enzymes. The induction of the cGAS-STING pathway was also observed in cells carrying TDP-43^G376D^, possibly leading to neuroinflammation. These results provide a deeper knowledge of molecular mechanisms responsible for ALS onset and progression, and could help to identify new therapeutic strategies.

## Data Availability

The original contributions presented in this study are included in the article. Further inquiries can be directed to the first, last or corresponding authors.

## Abbreviations

The following abbreviations are used in this manuscript:

ALS	Amyotrophic lateral sclerosis
ATP5A	ATP synthase lipid-binding protein
cGAS	Cyclic GMP-AMP synthase
COX2	Cytochrome oxidase activity
CTCF	Corrected total cell fluorescence
DMEM	Dulbecco’s modified eagle medium
DRP1	Dynamin-related protein 1
DTT	Dithiothreitol
ETC	Electron transport chain
FBS	Fetal bovine serum
FCCP	Carbonyl cyanide-4-(trifluoromethoxy)phenylhydrazone
FIS1	Mitochondrial fission 1 protein
GFP	Green fluorescent protein
GPX-1	Glutathione peroxidase 1
GPX-4	Glutathione peroxidase 4
HRP	Horseradish peroxidase
MFN2	Mitofusin 2
MMP	Mitochondrial membrane potential
MTCOI	Mitochondrial cytochrome C oxidase subunit I
ND3	NADH dehydrogenase 3
ND6	NADH dehydrogenase 6
NDUFS3	NADH: Ubiquinone oxidoreductase core subunit S3
NRF-1	Nuclear respiratory factor 1
OCR	Oxygen consumption rate
OPA1	Optic atrophy 1
PBS	Phosphate buffered saline
PCA	Perchloric acid
PGC1α	Peroxisome proliferator-activated receptor-gamma coactivator
PVDF	Polyvinylidene fluoride
RCR	Respiratory control ratio
ROS	Reactive oxygen species
SDHB	Succinate dehydrogenase B
SEM	Standard error mean
SOD2	Superoxide dismutase 2
STING	Stimulator of interferon genes
TARDBP	TAR DNA binding protein
TFAM	Mitochondrial transcription factor A
TMRE	Tetramethylrhodamine ethyl ester perchlorate
TOM20	Translocase of outer mitochondrial membrane 20
UQCRC2	Ubiquinol–cytochrome c reductase core protein 2
